# Meta-Analysis of the Efficacy and Safety of Ketamine on Postoperative Catheter-Related Bladder Discomfort

**DOI:** 10.3389/fphar.2022.816995

**Published:** 2022-06-27

**Authors:** Youyi Lu, Qi Li, Yunqiang Wang, Zhongbao Zhou, Dongxu Zhang, Yiping Bao, Jitao Wu, Yuanshan Cui

**Affiliations:** ^1^ Department of Urology, the Affiliated Yantai Yuhuangding Hospital of Qingdao University, Yantai, China; ^2^ Department of Endocrinology, Yantai City Municipal Government Hospital, Yantai, China; ^3^ Yantai Traditional Chinese Medicine Hospital, Yantai, China; ^4^ Department of Urology, Beijing Tian Tan Hospital, Capital Medical University, Beijing, China

**Keywords:** ketamine, catheter-related bladder discomfort, randomized controlled trials, CRBD, meta-analysis

## Abstract

**Objectives:** We conducted meta-analysis to demonstrate the efficacy and safety of ketamine on postoperative catheter-related bladder discomfort (CRBD).

**Methods:** A systematic search was performed through PubMed, Embase, and Cochrane Library to identify all randomized controlled trials that used ketamine in postoperative CRBD. This study was carried out by using the Preferred Reporting Items for Systematic Reviews and Meta-Analyses. We used RevMan version 5.3.0. to analyze the data.

**Results:** Five RCTs involving 414 patients were included in the analysis. The incidence and severity of postoperative CRBD were assessed at 0, 1, 2, and 6 h. According to our results of meta-analysis, ketamine reduced the incidence of postoperative CRBD at 2 h (RR 0.39; 95% CI, 0.21–0.71; *p* = 0.002, I^2^ = 40%) and 6 h (RR 0.29; 95% CI, 0.16–0.50; *p* < 0.0001, I^2^ = 0%) significantly; however, there were no statistical differences at 0 h (RR 0.81; 95% CI, 0.35–1.88; *p* = 0.62, I^2^ = 96%) and 1 h (RR 0.57; 95% CI, 0.13–2.54; *p* = 0.46, I^2^ = 97%). In two studies, we compared the incidence of moderate-to-severe CRBD between groups according to the scaling system (none, mild, moderate, and severe), and data are presented as numbers. Patients in the ketamine group showed a significantly lower severity of CRBD than those in the placebo group at 1 h (RR 0.09; 95% CI, 0.03–0.31; *p* = 0.0001) and 2 h (RR 0.06; 95% CI, 0.01–0.44; *p* = 0.005). In contrast, there were no meaningful differences between the two groups in the severity of CRBD at 0 h (RR 0.18; *p* = 0.84) or 6 h (RR 0.20; 95% CI, 0.03–1.59; *p* = 0.13). There were no meaningful differences on the rate of adverse events between the ketamine group and control group, mainly including postoperative nausea and vomiting (RR 1.24; 95% CI, 0.89–1.72; *p* = 0.21), diplopia (RR 3.00; 95% CI, 0.48–18.67; *p* = 0.24), and hallucination (RR 3.00; 95% CI, 0.32–28.24; *p* = 0.34).

**Conclusion:** Our meta-analysis demonstrated that a sub-hypnotic dose of ketamine administration can reduce the incidence and severity of postoperative CRBD without causing evident side effects.

## Introduction

Indwelling urinary catheter during surgery is common in order to ensure postoperative bladder drainage. However, patients with a urinary catheter during surgery often complained about discomfort in the supra-pubic region or a burning sensation of the urethra and urinary frequency with or without urgency incontinence. These symptoms are described as postoperative catheter-related bladder discomfort (CRBD), which extremely reduces patient quality of life (Agarwal et al., 2005; Li et al., 2016).

CRBD is similar to symptoms of overactive bladder (OAB) (Binhas et al., 2011). Both CRBD and OAB are considered to be associated with involuntary contractions of the bladder smooth muscle mediated by muscarinic receptors directly (Anderson, 1993; Agarwal et al., 2005; Agarwal et al., 2006a). The muscarinic receptor antagonist ketamine has been proven successful for CRBD (Durieux, 1995; Hirota and Lambert, 1996). Ketamine, a phenylic dine derivative, is a general anesthetic drug. In recent years, there has been more interest in the use of ketamine as a perioperative analgesic (Adam et al., 2005; Loftus et al., 2010; Mueller and Golembiewski, 2011). Besides muscarinic receptors, ketamine also interacts with many other receptors, including N-methyl-d-aspartate (NMDA) receptors and opioid receptors (Kohrs and Durieux, 1998). The interaction mechanisms are variable and complex. Clinically, intravenous (IV) infusion is the most common route of ketamine administration. Although several studies have been published to verify the efficacy and safety of ketamine on postoperative CRBD, there is a lack of meta-analysis to identify the conclusion. Therefore, we conducted a meta-analysis of randomized controlled trials (RCTs).

## Materials and Methods

### Search Strategy and Criteria

A systematic search was performed through PubMed, Embase, and Cochrane Library to identify all studies published until October 2021. The following search terms were applied for the search: RCT, ketamine, and CRBD.

### Inclusion Criteria Were

(a) Randomized controlled trials;
(b) The effect of ketamine on postoperative CRBD was studied, and the route of administration of ketamine is intravenous infusion;
(c) The placebo is saline;
(d) Full text and related data can be available;
(e) Articles published in English.

All authors independently browsed and read all searched articles, and the final list of included articles was decided through a consensus discussion.

### Data Extraction

Data extraction was performed independently by all authors. Disagreements were resolved by consensus. The following characteristics were included: the primary author, publication year, catheter size, types of surgery, a dose of ketamine, duration of operation, primary and secondary the outcome, and time point of ketamine administration, as shown in Table 1.

**TABLE 1:** Characteristics of included studies.

Study	Dose of ketamine	Gender (Male/Female)		Catheter Size	Types of surgery	Primary outcome	Secondary outcome	Check points	Time	Duration of operation (mean ± SD, min) k/c
		Ketamine(K)	Control (C)							
Agarwal 2006 (Agarwal et al., 2006b)	0.25 mg/kg I.V.	23/2	24/1	16 Fr	elective percutaneous nephrolithotomy	Incidence of CRBD severity of CRBD	Side-effects	0 h, 1 h, 2 h, and 6 h	After the occurrence of CRBD	112.4 ± 34.7 vs. 105.6 ± 28.8
Shariat Moharari 2014 (Shariat Moharari et al., 2014)	0.5 mg/kg I.V.	54/3	53/4	16 Fr	elective nephrectomy	Incidence of CRBD severity of CRBD	Side-effects	0 h, 1 h, 2 h, and 6 h	Before the occurrence of CRBD	102 ± 18 vs. 99 ± 21
Safavi 2014 (Safavi et al., 2014)	0.25 mg/kg I.V.	20/10	21/9	16 Fr	elective urologic surgery requiring catheterization of the urinary bladder	severity of CRBD	Sedation score (0–4) Rescue analgesic (mg) Adverse effects	15, 30, and 45 min, 1 h, 2 h, 6 h, 12 h, and 24 h	After the occurrence of CRBD	61 ± 15 vs. 62 ± 24
Akca 2016 (Akça et al., 2016)	0.25 mg/kg I.V.	NG	NG	NG	Undergoing cystoscopy	Incidence of CRBD severity of CRBD	Side effects (PONV)	0 h, 15 min, 1, 2, and 6 h	Before the end of surgery	NG
Burimsittichai 2016 (Burimsittichai et al., 2016)	0.5 mg/kg I.V.	14/56	14/56	14 or 16 Fr	elective laparoscopic surgery	severity of CRBD	Pain intensity Sedation rate	0 h, 1 h, 6 h, and 24 h	Before urinary catheterization	117 ± 49.4 vs. 113.8 ± 55.1

NG: not given in the article.

### Statistical Analysis

The abstracted data were calculated by using Rev Man version 5.3.0 (The Cochrane Collaboration, London, United Kingdom). Variables were pooled only if evaluated by ≧2 studies. The mean difference (MD) with 95% confidence intervals (CIs) was utilized to analyze the continuous data, and the risk ratios (RRs) with 95% CIs were applied to analyze the dichotomous data among the different groups. A fixed-effect model was used as there was no significant heterogeneity (*p*-value of x^2^ test no less than 0.10 and I^2^ not greater than 50%); otherwise, a random-effects model was used (*p*-value of x^2^ test less than 0.10 and I^2^ greater than 50%). A *p* value of less than 0.05 was considered statistically significant.

### Quality Assessment

**FIGURE 1:**
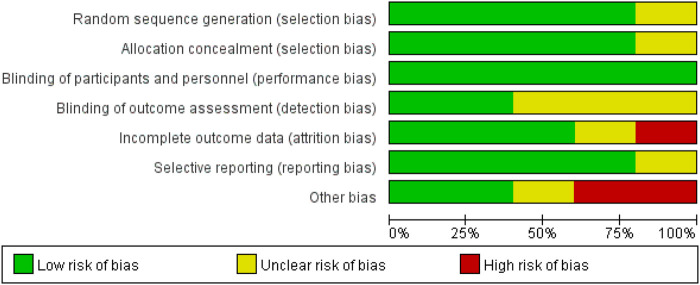
Risk of bias graph.

**FIGURE 2:**
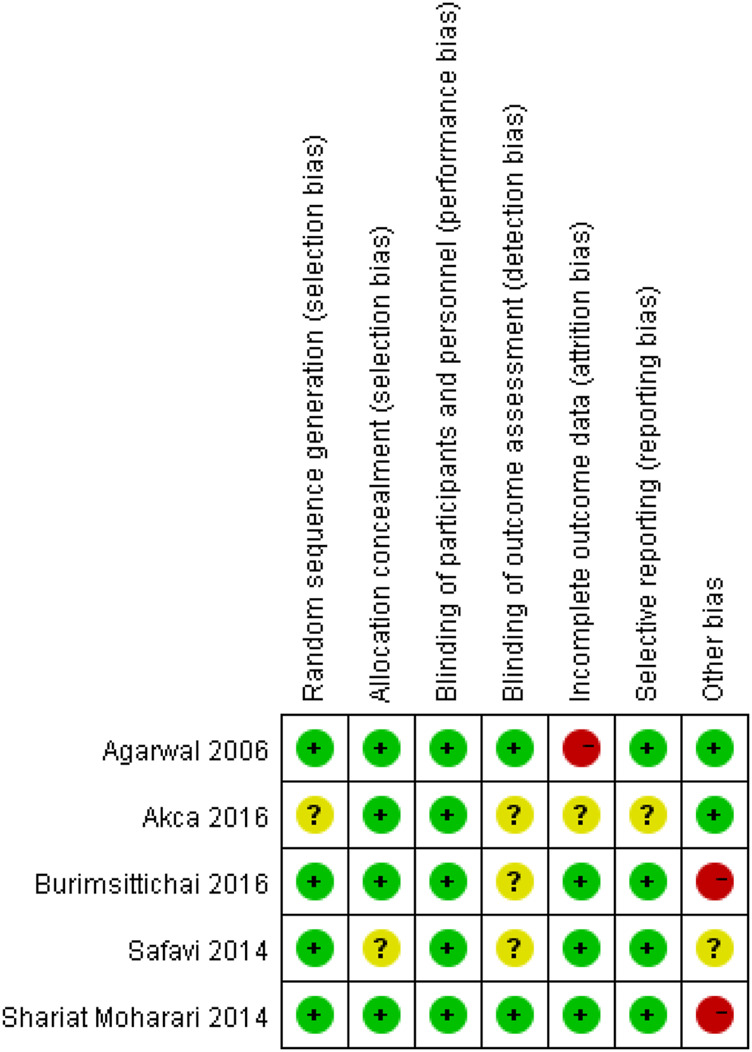
Risk of bias summary.

We used the Cochrane Risk of the bias assessment tool and assigned assessments of low, high, or unclear risk of bias (Higgins and Green, 2011). Figure 1 and Figure 2 demonstrated an overview of the risk of bias.

## Results

**FIGURE 3:**
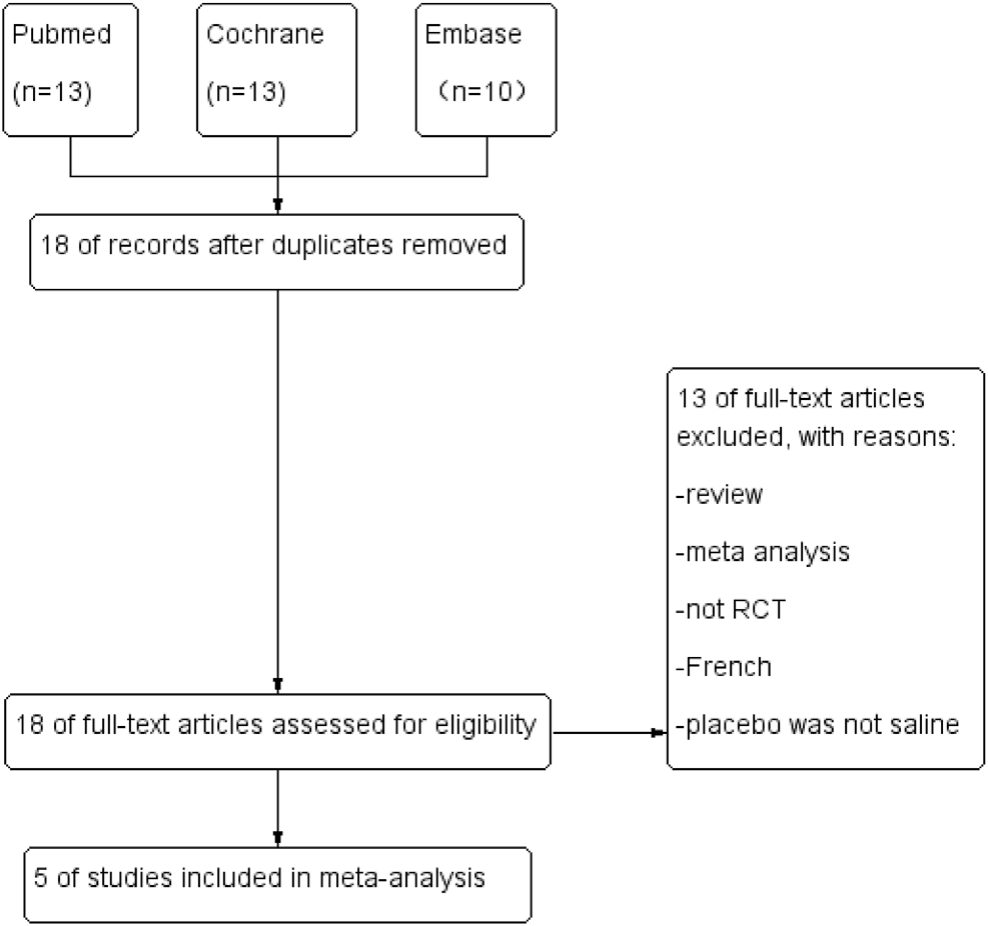
Flow diagram.

We obtained 18 relevant studies through a systematic search. After carefully reviewing the full text, 13 studies were excluded owing to the various reasons described in Figure 3. Figure 3 summarized the total flowchart. Finally, five RCTs (Agarwal et al., 2006b; Safavi et al., 2014; Shariat Moharari et al., 2014; Akça et al., 2016; Burimsittichai et al., 2016) involving 414 patients were included in the meta-analysis.

### Characteristics of Studies

All patients in the included studies were treated with 16G or 18G Foley catheters. The patient characteristics in the two groups were similar with respect to age (MD 0.75, 95% CI -1.16 to 2.65, *p* = 0.44), weight (MD -0.24, 95% CI -4.02 to 3.53, *p* = 0.90), sex ratio (RR 0.99, 95% CI 0.90 to 1.09, *p* = 0.83), and duration of surgery (MD 2.27, 95% CI -3.03 to 7.56, *p* = 0.40). The dose of ketamine in the included studies was 0.25 mg/kg ((Agarwal et al., 2006b), (Safavi et al., 2014), and (Akça et al., 2016)) and 0.5 mg/kg ((Shariat Moharari et al., 2014) and (Burimsittichai et al., 2016)).

### Incidence of Postoperative CRBD

**FIGURE 4:**
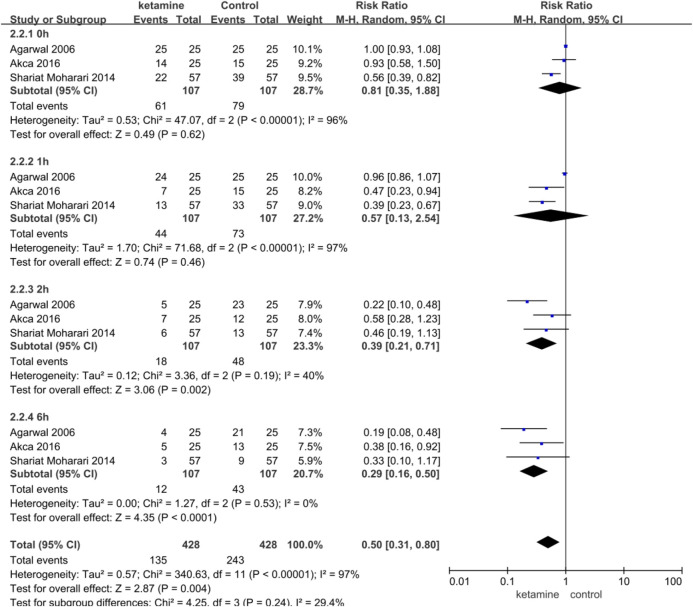
Incidence of catheter-related bladder discomfort in ketamine vs. placebo.

Three studies enrolling 214 participants (107 in the ketamine group and 107 in the control group) were used to analyze the impact of ketamine on the incidence of postoperative CRBD. Ketamine reduced the incidence of postoperative CRBD at 2 h (RR 0.39; 95% CI, 0.21–0.71; *p* = 0.002, I^2^ = 40%) and 6h (RR 0.29; 95% CI, 0.16–0.50; *p* < 0.0001, I^2^ = 0%) significantly; however, there were no statistical differences at 0 h (RR 0.81; 95% CI, 0.35–1.88; *p* = 0.62, I^2^ = 96%) and 1 h (RR 0.57; 95% CI, 0.13–2.54; *p* = 0.46, I^2^ = 97%) (Figure 4).

### The Severity of Postoperative CRBD

**FIGURE 5:**
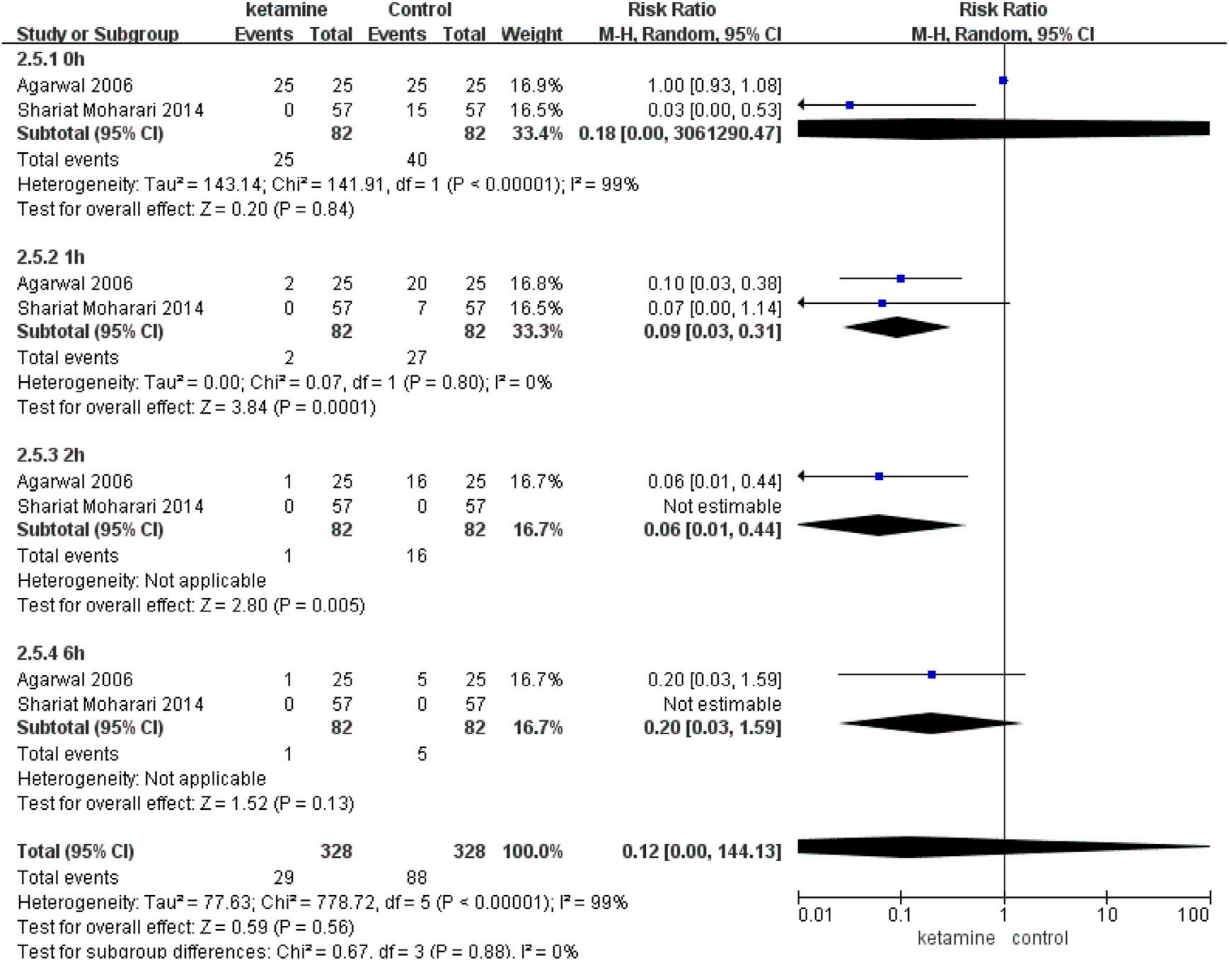
Incidence of moderate-to-severe catheter-related bladder discomfort in ketamine vs. placebo.

In two studies (Agarwal et al., 2006b; Shariat Moharari et al., 2014) (82 in the ketamine group and 82 in the control group), we compared the incidence of moderate-to-severe CRBD between groups according to the scaling system (none, mild, moderate, and severe), and data are presented as numbers. Patients in the ketamine group showed a significantly lower severity of CRBD than those in the placebo group at 1 h (RR 0.09; 95% CI, 0.03–0.31; *p* = 0.0001) and 2 h (RR 0.06; 95% CI, 0.01–0.44; *p* = 0.005). In contrast, there were no meaningful differences between the two groups in the severity of CRBD at 0 h (RR 0.18; *p* = 0.84) or 6 h (RR 0.20; 95% CI, 0.03–1.59; *p* = 0.13) (Figure 5).

### Safety

**FIGURE 6:**
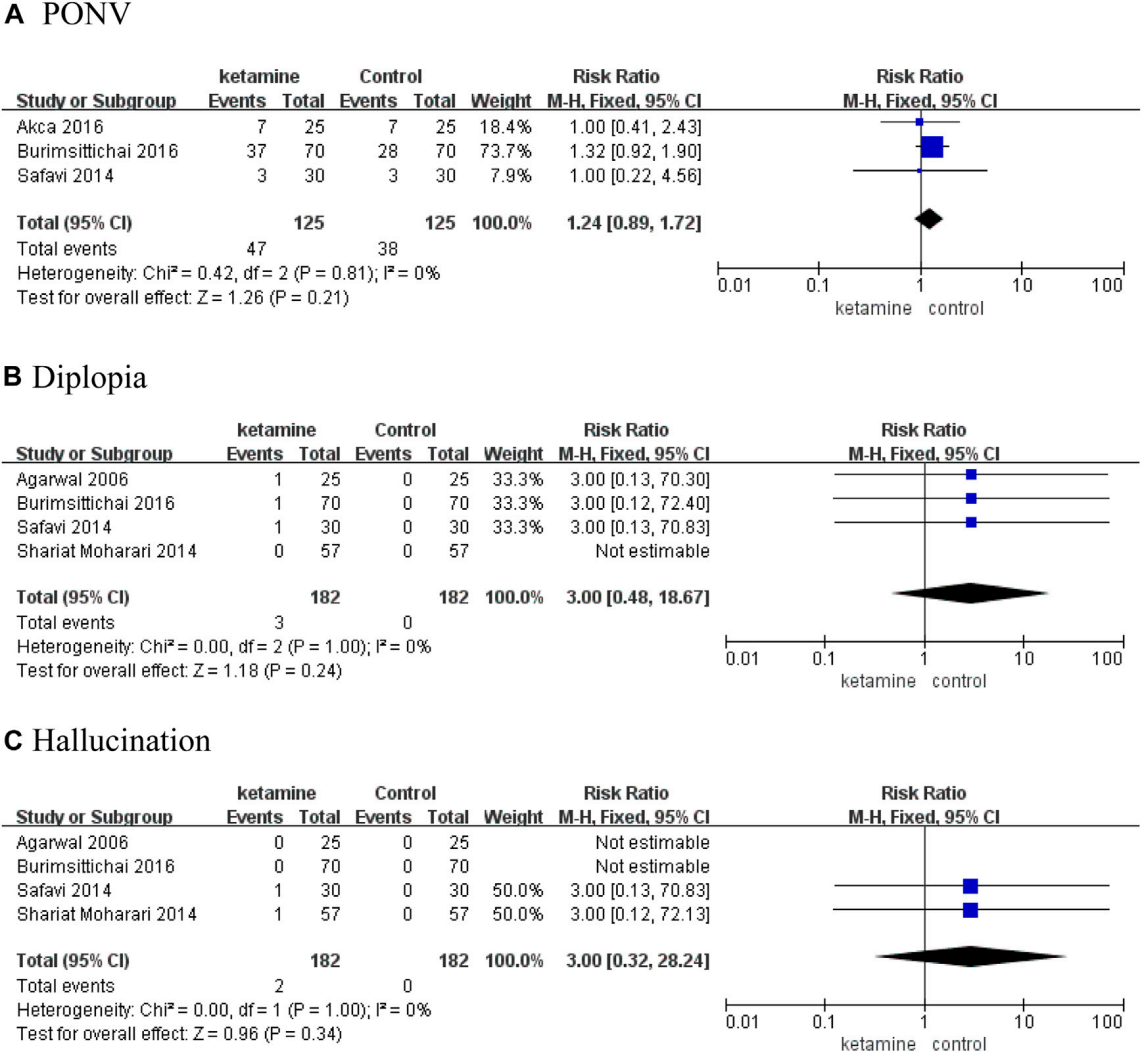
Forest plot of adverse events. **(A)** PONV. **(B)** Diplopia. **(C)** Hallucination.

There were no meaningful differences on the rate of adverse events between the ketamine and control groups, mainly including postoperative nausea and vomiting (PONV) (RR 1.24; 95% CI, 0.89–1.72; *p* = 0.21), diplopia (RR 3.00; 95% CI, 0.48–18.67; *p* = 0.24), and hallucination (RR 3.00; 95% CI, 0.32–28.24; *p* = 0.34) (Figure 6).

## Discussion

Urinary catheterization is a necessary procedure in most surgeries. CRBD is secondary to an indwelling urinary catheter, which is characterized by the symptoms of urinary frequency and urgency in the overactive bladder and an urge to void or discomfort at the supra-pubic region (Hu et al., 2016). The incidence of CRBD has been reported to range from 47 to 90% (Binhas et al., 2011). CRBD is common in surgeries requiring postoperative catheterization, especially urologic surgery. Postoperative CRBD may increase postoperative complications, prolong hospital stay, and reduce the quality of recovery.

The bladder expresses many muscarinic receptors (a majority of M2 muscarinic receptor subtype and a few of M3 receptors). Activation of the M2 receptor causes the contraction of the detrusor smooth muscles; whereas selective M3 receptor inactivation results in M2-mediated contraction of the detrusor muscle (YamanishiChapple et al., 2001). Muscarinic receptor antagonists were investigated to treat CRBD. Currently, many antimuscarinic reagents, including ketamine, have been used successfully for the treatment of CRBD (Andersson, 1999; Agarwal et al., 2007; Tauzin-Fin et al., 2007; Bala et al., 2012; Ryu et al., 2013; Zhang et al., 2014).

Ketamine is well-received because of its unique properties such as protection of the upper respiratory tract reflex, without significant respiratory inhibition, and its effective analgesic effect (Mion and Villevieille, 2013; Persson, 2013). Ketamine, a dissociative anesthetic, has an analgesic effect in sub-anesthetic doses. Ketamine is a complex medication with two isomers, R (-)-ketamine and S (+)- ketamine (Sinner and Graf, 2008). Ketamine could interact with many receptors, including opioid receptors, NMDA receptors, and muscarinic receptors (Kohrs and Durieux, 1998). The activation of the NMDA receptor leads to an influx of Ca^2+^ closely involved in the development of central sensitization of dorsal horn neurons (Quibell et al., 2011), which plays an important role in pain sensation. Ketamine acts by reducing the frequency and opening of the Ca^2+^ channel and also prevents Ca^2+^ influx by antagonizing the NMDA receptor noncompetitively (Orser et al., 1997; Fan et al., 2012).

The bioavailability of oral ketamine is poor. Due to the extensive first-pass elimination effect, only 17% of an oral dose is absorbed (Clements et al., 1982). At present, there are no FDA-approved nonparenteral preparations for oral administration. Intravenous injection is the main medication administration in postoperative pain. IV ketamine gets an advantage because it takes effect quickly (within 30 s and the maximum effect occurs in about 1 min).

Ketamine is dose-dependent. According to the United States Food and Drug Administration (FDA) prescribing information, the average dose is 2 mg/kg as induction of anesthesia. However, the anesthetic dose is considered undesirable because it may produce prolonged emergence and unpleasant side effects. Ketamine is recommended for use in sub-anesthetic doses to provide adequate analgesia (Slogoff et al., 1974). The Consensus Guidelines on the Use of Intravenous Ketamine Infusions for Acute Pain Management recommends lower doses (0.1–0.5 mg/kg per hour) in acute pain therapy to achieve an adequate balance of analgesia and adverse effects (Schwenk et al., 2018). The common sub-anesthetic dose of IV ketamine used in clinical practice is 0.25 and 0.5 mg/kg.

At present, a sub-hypnotic dose of ketamine has been used in pain management. The pharmacokinetics of ketamine has been widely reported. It was reported that the mean serum half-life of ketamine was about 2–3 h (Reves et al., 2010; Clements and Nimmo, 1981; Idvall et al., 1979; Wieber et al., 1975). In our meta-analysis, five RCTs involving 414 patients were included. The incidence and severity of CRBD after surgery were assessed at 0, 1, 2, and 6 h. According to our results, ketamine IV administration could reduce the incidence of postoperative CRBD significantly at 2 and 6 h and reduce the severity of CRBD at 1 and 2 h compared with the control group. Our results are consistent with the pharmacokinetics of ketamine. In the early stage after surgery, ketamine can reduce the severity of CRBD because the maximum plasma concentration can be reached rapidly. With prolonged postoperative observation time, the incidence of CRBD is also reduced.

The probable side effects reported for ketamine were PONV, sedation, diplopia, hallucination, unpleasant dreams, and respiratory depression. We analyzed PONV, hallucination, and diplopia according to the data of the included RCTs. The results showed ketamine was safe without evident side effects. It is worth mentioning that sedation is a more important side effect for sub-anesthetic dose of ketamine. However, although four articles (Agarwal et al., 2006b; Shariat Moharari et al., 2014; Akça et al., 2016; Burimsittichai et al., 2016) in our meta-analysis showed information on the side effects of sedation, the data were still insufficient and the sedation scales used in the enrolled literature were different. We finally obtained the data on moderate and severe sedation in two studies ((Shariat Moharari et al., 2014) and (Burimsittichai et al., 2016)). The results showed statistically significant differences in terms of moderate and severe sedation (RR 7.67; 95% CI, 1.42–41.39; *p* = 0.02). However, because there are only two included articles, the conclusion needs to be further verified in the future.

In addition, we think urologists need to know about ketamine-related cystitis which is also a serious problem in the clinic. The United States Food and Drug Administration (FDA) prescribing information shows in individuals with a history of chronic ketamine use or abuse that there have been case reports of genitourinary pain that may be related to the ketamine treatment, not the underlying condition (Schwenk et al., 2018). Ketamine cystitis was first reported in a case series in 2007 and is characterized by severe dysuria, urgency, frequency, and gross hematuria (Shahani et al., 2007). The pathophysiology of ketamine cystitis remains unclear. It was reported that there is a dose and frequency response relationship between ketamine use and urinary symptoms, and 51% of patients had improved their symptoms after stopping ketamine usage (Winstock et al., 2012), therefore considering the cessation of ketamine if genitourinary pain continues in the setting of other genitourinary symptoms.

There are several limitations in our analysis: 1) the number of included studies was small. Therefore, subgroup analysis or sensitivity analysis could not be conducted further. In our opinion, the lack of industry interest in funding large and multicenter studies, as well as the ethical and practical concerns related to enrolling patients with acute pain conditions in controlled clinical trials, may be the main reasons for the insufficient number of clinical trials. However, we believe that our meta-analysis is meaningful and can provide new information for doctors in reducing postoperative CRBD. At the same time, we also hope there will be more RCTs with larger sample sizes and high quality for a better understanding of ketamine for the treatment of CRBD in the future; 2) significant heterogeneity exists in our analysis, which may introduce a bias; 3) type of surgery may result in potential bias. Only one study included in the meta-analysis was elective laparoscopic surgery, while the other four studies were all urology surgeries. We could not make a further subgroup analysis because of a small number of studies; 4) it is worth mentioning that the time point of ketamine administration in our analysis is inconsistent which may contribute to the high heterogeneity. In our study, we did not assess the dose–response effects and timing of ketamine administration, which can be a very important variable in outcome analysis.

In conclusion, we suggest that a sub-hypnotic dose of ketamine administration decreases the incidence and severity of postoperative CRBD. In addition, a sub-hypnotic dose of ketamine is safe for postoperative CRBD. More RCTs with larger sample sizes and high quality are needed for a better understanding of a sub-hypnotic dose of ketamine for the treatment of CRBD.

## Conclusion

Our meta-analysis demonstrates that a sub-hypnotic dose of ketamine decreases the incidence and severity of postoperative CRBD without causing evident side effects. More experimental studies are needed to confirm the causality. Further research is worthwhile regarding the timing or dose of ketamine administration.

## Data Availability

The original contributions presented in the study are included in the article/Supplementary Material; further inquiries can be directed to the corresponding authors.

## Abbreviations

CRBD: catheter-related bladder discomfort; OAB: overactive bladder; NMDA: N-methyl-d-aspartate; IV: intravenous; RCTs: randomized controlled trials; MD: mean difference; RR: risk ratio; CI: confidence interval; NG, not given in the article.
